# Rheological Properties of Nine Commercially Available Endodontic Sealers by Substrate and Temperature: A Comparative In Vitro Study

**DOI:** 10.3390/jfb17090448

**Published:** 2026-09-05

**Authors:** George G. Sotiropoulos, Michael Solomonov, Konstantinos Koutoulas, Nikos Pantazis, Eleftherios Terry Farmakis

**Affiliations:** 1 Private Practice Limited in Endodontics, 12351 Agia Varvara, Greece; gsgsotirop@gmail.com; 2 Department of Endodontics, Israel Defense Forces (IDF) Medical Corps, Tel Hashomer, Ramat Gan 5552507, Israel; mishasol@gmail.com; 3 Private Practice Limited in Endodontics, 20100 Corinth, Greece; konktls@gmail.com; 4 Department of Hygiene, Epidemiology and Medical Statistics, School of Medicine, National and Kapodistrian University of Athens, 11527 Athens, Greece; npantaz@med.uoa.gr; 5 Department of Endodontics, Dental School, National and Kapodistrian University of Athens, 11527 Athens, Greece

**Keywords:** endodontic sealer, rheological properties, temperature, dentin, gutta-percha

## Abstract

Background: This study aimed to investigate flow, contact angles and capillary penetration of commercially available endodontic sealers by substrate (human root dentin discs [HRD] and gutta-percha [GP]) and by temperature [room (20 °C), or body (37 °C)]. Methodology: Nine commercially available endodontic sealers were tested using the two-plate method, contact angle calculation (measured on HRD and GP surface—for both temperatures), free-flow, and penetration co-efficiency using a capillary permeability test (in 37 °C). Discs, set-ups and sealers were prepared according to the protocol; specimens were photographed; results were collected; and all recordings were statistically analyzed. Results: The chemical composition of each sealer significantly affected rheological properties even in the same chemical group (*p* < 0.001). Also, recordings from every test made were also significantly influenced by temperature (*p* < 0.001). More specifically, the behaviour of sealers under higher temperature proved to be sealer-specific. Conclusions: Within the limitation of this study, it was verified that Endodontic sealers’ rheological properties were affected by the substrate’s composition and temperature. Additionally, body temperature significantly influenced thier performance.

## 1. Introduction

A root canal sealer is as a radiopaque dental cement that is used, usually in combination with a solid or semisolid core material, to fill voids and seal root canals during obturation [1]. The success of root canal treatment relies on the complete cleaning of the root canal system, the elimination of bacteria, and the hermetic seal of the root canal [2]. An adequate root canal seal cannot be achieved without the presence of a root canal sealer, because gutta-percha does not adhere to root canal walls [3]. Also, pulp space irregularities cannot be sealed predictably with gutta-percha; therefore the sealer is expected to adapt to the local anatomy and serve as filling material [4].

Grossman’s suggested properties of an ideal root canal sealer [5], in clinical practice, are less relevant if the sealer does not flow into such irregularities due to its physical properties [6]. In this case, there will be space for pathogens to survive—particularly through leakage—affecting healing [7]. Ideally, a root canal sealer should contribute significantly to three-dimensional obturation throughout the canal by promoting adhesion between the gutta-percha and root canal walls, thereby minimizing gaps at the sealer–dentine interface and, thus, leakage [8,9,10,11,12].

Successful obturation is obtained through the adhesion and stability of the sealer to the canal walls, which, among other factors, is influenced significantly by its rheological properties, such as viscosity and flow [13]. Rheology originates from the Greek ancient word ῥοή, which means flow and/or movement of matter; thus, it is the scientific study of how materials deform and flow in response to applied forces (this also includes their own weight), encompassing both solid-like and fluid-like behaviours, and linking these responses to the material’s properties and performance [14]. 

Among such properties that dictate the suitability of a root canal sealer are adequate flow rate and wetting [15,16]. Adequate flow is a clinically significant factor that must be realized to achieve deep penetration into the narrow and complex anatomical spaces of the root canal system [3]. 

Wetting is the interface contact between a liquid and solid, accompanied by the simultaneous expulsion of air. Contact angle measurements can be used to increase our understanding of the interactions between solids and liquids. These interactions are central in examining not only the wettability of a material but also the wetting, spreading, and adsorption of liquids. Lower contact angle values entail faster spreading of the sealer to dentin substrate and gutta-percha surfaces [17].

Despite the variety of existing sealers, the ideal compound has not been identified. Sealers vary in composition to enhance their physical characteristics. The impact of compositional modifications on clinically important properties, such as flow, has not been examined extensively [18].

Today, various types of endodontic sealers are available, including sealers that are based on zinc oxide—such as zinc oxide eugenol (ZnOE)—and resin-based (epoxy or UDMA), glass ionomer, calcium hydroxide, silicone, mineral trioxide aggregate (MTA)-based (which is actually Salicylate) and calcium silicate root canal sealers.

The aim of this study was to compare some rheological properties, and to be exact: free and under pressure flow, contact angles, and capillary penetration of nine commercially available endodontic sealers on two substrates (human root dentin and GP surfaces, under two different temperatures—bench and body temperature) and find out how these compare to those suggested by the ANSA/ADA and ISO directives.

## 2. Materials and Methods

The sealers included in this study, are shown in Table 1:

The experimental setups used in this study were:

(1) Viscosity-related measures of diameter in a 2-plate system
(2) Comparison of wetting by contact angles test of these root canal sealers, without the application of any load on root dentin and gutta-percha surfaces, over 4 periods (0, 1, and 5 and 60 min) and at 2 temperatures (20 °C and 37 °C). In vitro testing of these physical properties is a basic prerequisite for the introduction of any new material into clinical endodontic practice [19].
(3) Free-flow/lateral-flow/creeping/time-dependent deformation at body temperature (37 °C) through lateral-flow test using vertical glass plates, measured at 1 min.
(4) Determination of penetration coefficients by capillary permeability test at body temperature (37 °C), measured after 1 min.

There were 9 different sealers belonging to 5 different chemical structure families tested, in order to evaluate their performance even with the same chemical composition. The sealers that were tested in this study, listed in Table 1, were prepared and used according to the manufacturers’ instructions, prior to each test. There were two observers that were calibrated over non-included samples in all tests (Lin’s concordance correlation coefficient 0.96).

A priori sample-size planning was performed using G*Power version 3.1.7 (Heinrich Heine University Düsseldorf, Germany). Because the study included several experimental outcomes and factorial comparisons, the calculation was used as a general planning benchmark for experimental replication rather than as a separate power calculation for every final statistical model. A simplified repeated-measures planning scenario with nine material groups assumed a medium effect size (Cohen’s f = 0.25), α = 0.05, 80% power, a correlation of 0.50 among repeated measurements, and a nonsphericity correction of ε = 1; under these assumptions, approximately 8 experimental units per material/group were required. Actual replication depended on the assay design.

Study of flow

Two-plate method

**Figure 1:**
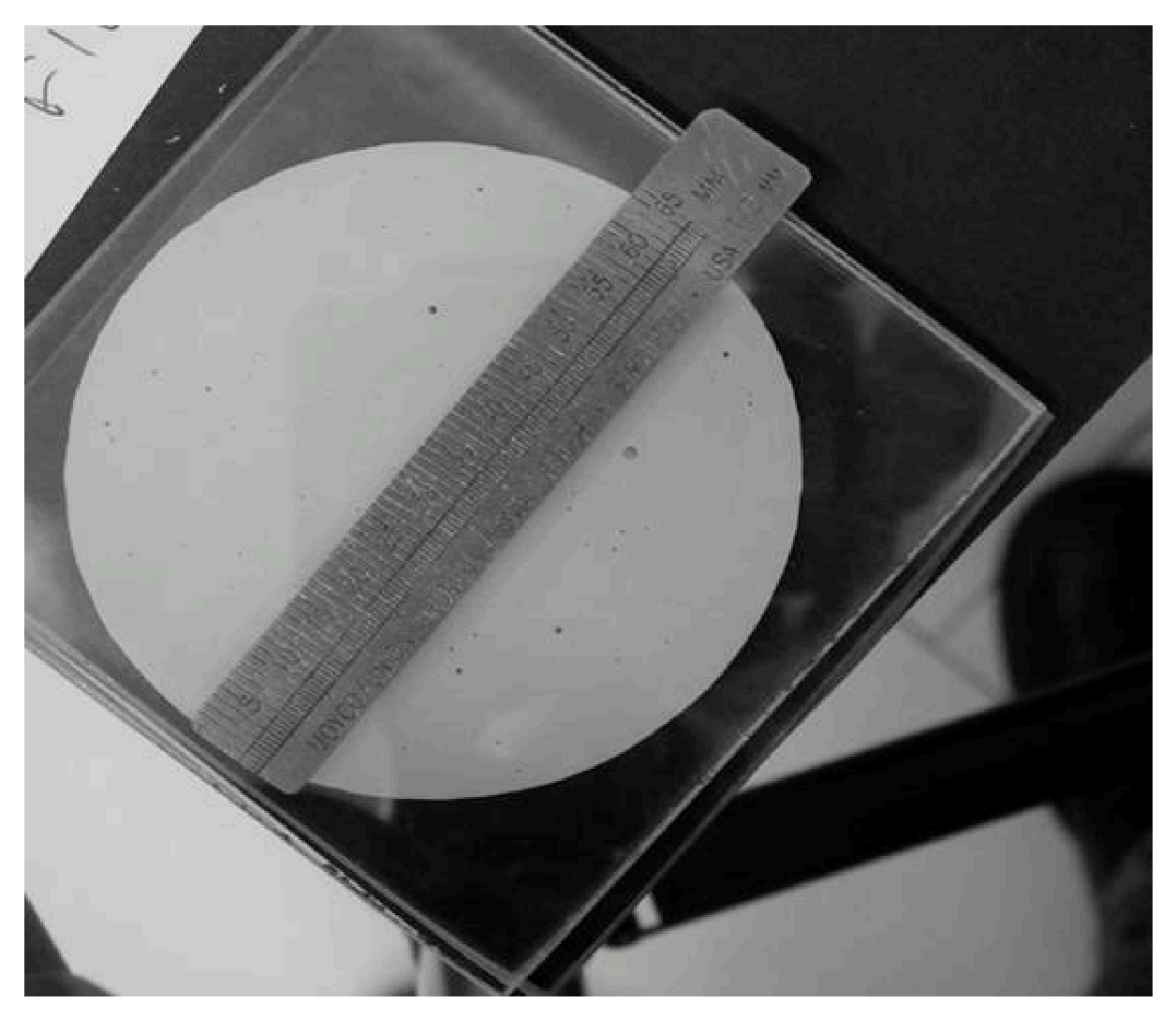
Two-plate method.

This experiment was performed at room temperature (20 ± 1 °C) and body temperature (37 ± 1 °C). Standardized square glass plates were produced to specifications by a glazier: 3 mm thickness, 4.5 mm square base, and weighing 120 g. The underlying plate was placed over marked borders, and the camera was secured in a vertical direction, to standardize recording conditions. A volume of 0.5 mL of each sealer was delivered via a 1 mL syringe onto the base plate, and then covered with the top plate, which was an identical 120 g glass plate. At 10 min, the minimum and maximum diameters of the resulting disc of each sample were measured at that moment using a digital micrometre (Swiss Precision Instruments, Los Angeles, CA, USA) with 0.01 mm resolution. The recorded measurements were verified by two independent investigators (GS and ETF). If the diameters were within 1.0 mm of each other, the mean of the 2 diameters was recorded; otherwise, the test was repeated. Additionally, a photograph was taken, with a metallic ruler on the diameter, for future reference and documentation. The flow of the sealer was determined, based on the mean of 6 [2-plate] tests that were performed for each sealer (Figure 1).

Study of wetting

Contact angles test

**Figure 2:**
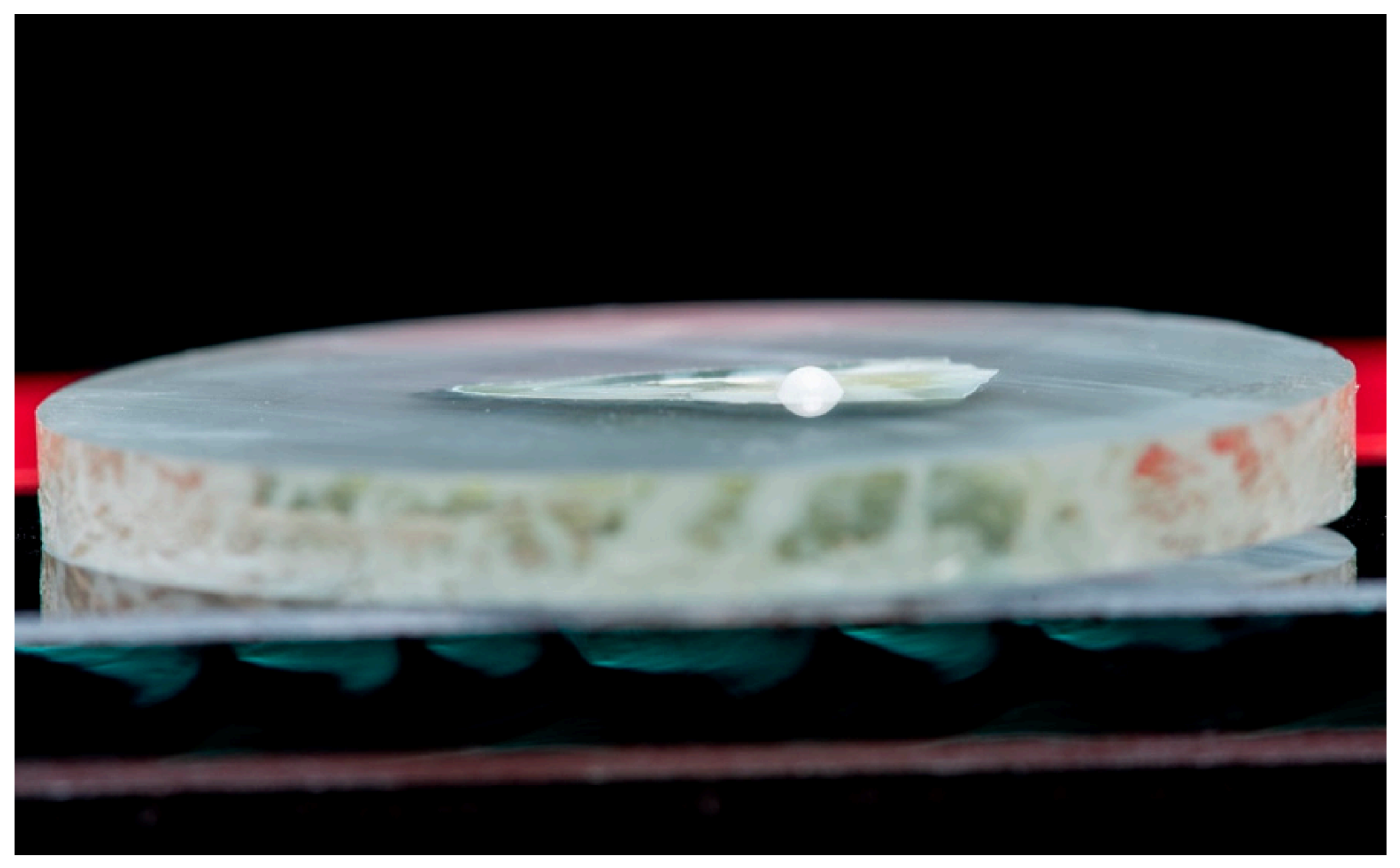
A dentin disc with a drop of sealer.

Two types of substrates were measured: (a) root dentin surfaces and (b) gutta-percha surfaces. Forty extracted, intact, fresh and caries-free maxillary molars, extracted for orthodontic, periodontal or prosthetic reasons, were used to prepare an equal number of root dentin discs. The study was conducted in accordance with the Declaration of Helsinki and approved by the Institutional Ethics Committee of National and Kapodistrian University of Athens (No 322/23/02/2017). The extracted teeth were rinsed thoroughly under running tap water and then placed in 10% formalin until use. After storage, the palatal roots were sectioned on the palatal side—along the root, 1 mm from the root canal, and 2 mm from the root canal to obtain oval sections of the root dentin surface (Figure 2). The roots were initially cut with a diamond disc (Komet; Brasseler GmbH, Lemgo, Germany) under running water. Polishing paper no. 0.5 was used to reduce the roughness of the dentin surfaces. The dentin surfaces were then bathed ultrasonically in distilled water for 5 min to rid them of any extraorganic components, after which the specimens were placed in an incubator (Cultura M, Almedica, Galmiz, Switzerland) for 5 min at 37 °C to dry. The dentin samples were transported directly from the incubator and placed into the measuring device.

With regard to the gutta-percha surfaces, 2 glass plates and thermoplasticized gutta-percha (Obtura Spartan, Fenton, MO, USA) were used to prepare 40 flat, smooth gutta-percha surfaces. 

Next, the root dentin and gutta-percha specimens were positioned one by one on a flat glass surface in the measuring device. A fixed volume of droplets (0.1 mL) of each sealer was placed onto 10 dentin discs and 10 gutta-percha surfaces. The volume of each sealer was controlled with a micropipette (Eppendorf Reference, adjustable-volume, Eppendorf AG, Hamburg, Germany). Each specimen was photographed twice [contra lighted (backlit another term, under standardized conditions)] at 0, 1, 5, and 60 min at 20 °C and 37 °C in a special incubator (Cultura M, Almedica, Switzerland) after the droplets were placed. The experiments were performed under standard conditions for temperature and relative humidity. The temperature was kept constant to within 1 °C with a thermostat in the incubator.

Photographs of the samples were taken with a fixed camera—to enable repeatable measurements—after the samples were placed in a standard-sized plastic box, and images of the droplets of each sealer were digitalized on a scanner. Next, the base width (b) and height (h) of the droplet meniscus were measured in SigmaScan Pro V 5.0.0 (1987–1999 SPSS, Inc., Leesburg, FL, USA). The contact angles were calculated per the equation

Θ^o^ = 2 arc [sin 2 h/b].

**Figure 3:**
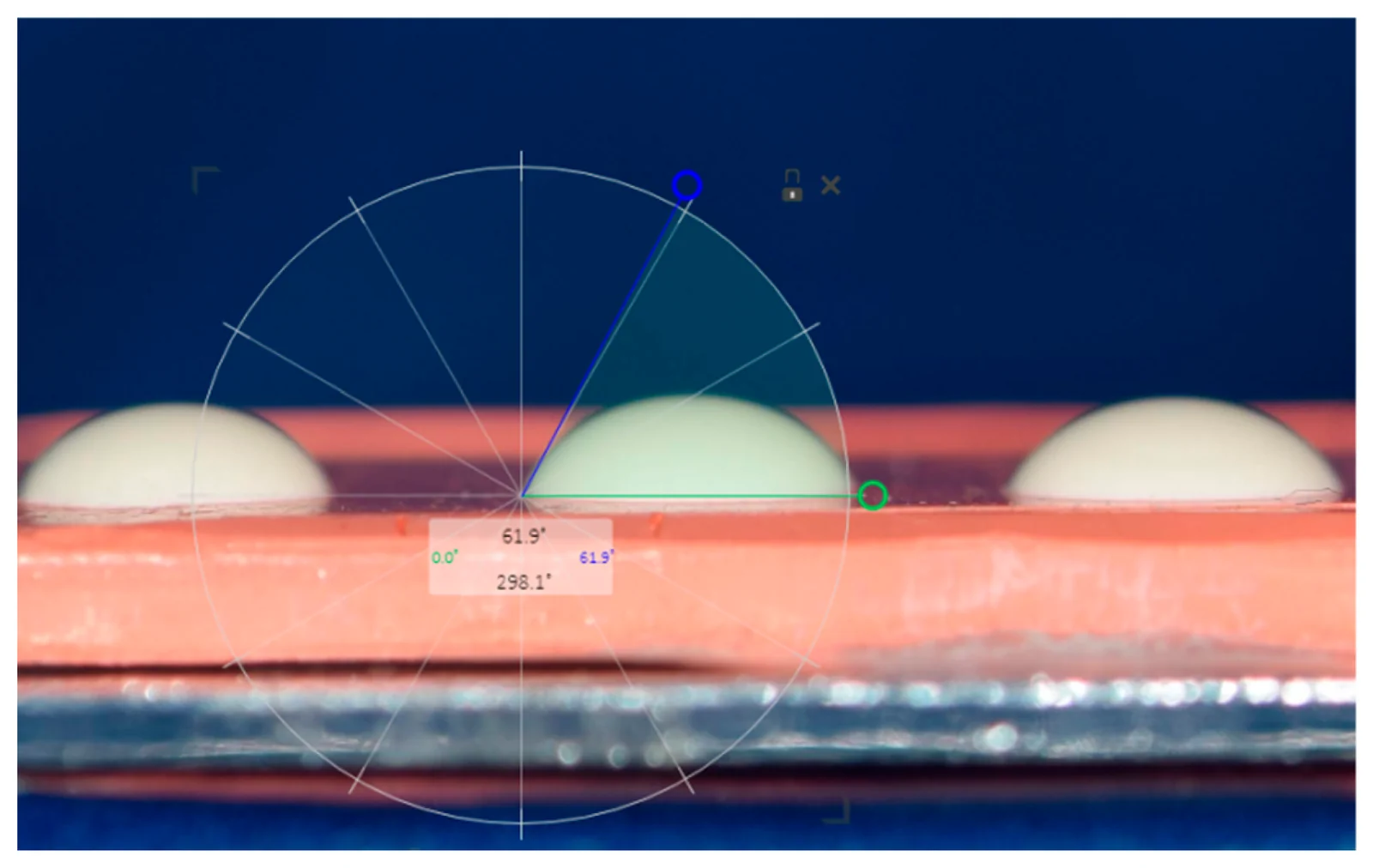
Calculation of contact angle of a sealer onto gutta-percha.

Figure 3 depicts the calculation of the contact angle of a sealer onto gutta-percha. All tests were two-sided, and the level of significance was set to 5% [20].

Penetration coefficient

Permeability in fine glass capillary tubes was selected as a suitable in vitro model for assessing flow in root canals. The distance that each given sealer travels inside the capillary tube in a given timeframe (30 s, in this case) reflects the potential of the sealer to penetrate fine spaces.

**Figure 4:**
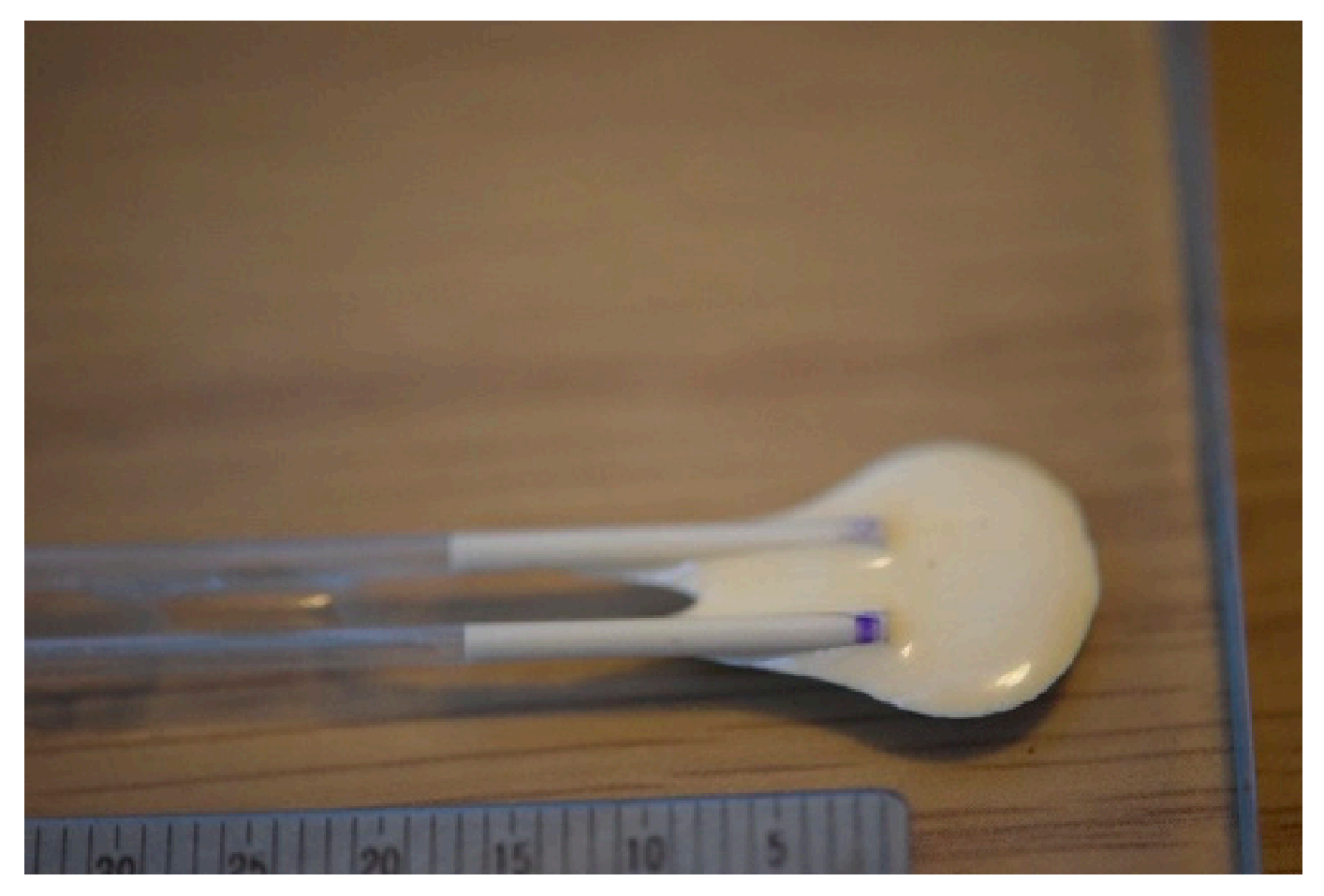
Sealer entered microcapillary tube.

This experiment was performed at simulated body temperature (37 ± 1 °C). Two microcapillary glass pipettes were fixed with glue to a glass plate that was 3 mm thick, with a 4.5 cm square base. One millilitre of each sealer was delivered with a micropipette (Eppendorf Reference, adjustable-volume, Eppendorf AG, Hamburg, Germany). After 30 s, the distance to which each sealer penetrated the microcapillary tube was measured and photographed, using the same setup as the two-plate method (Figure 4).

Lateral-flow/creeping/time-dependent deformation

**Figure 5:**
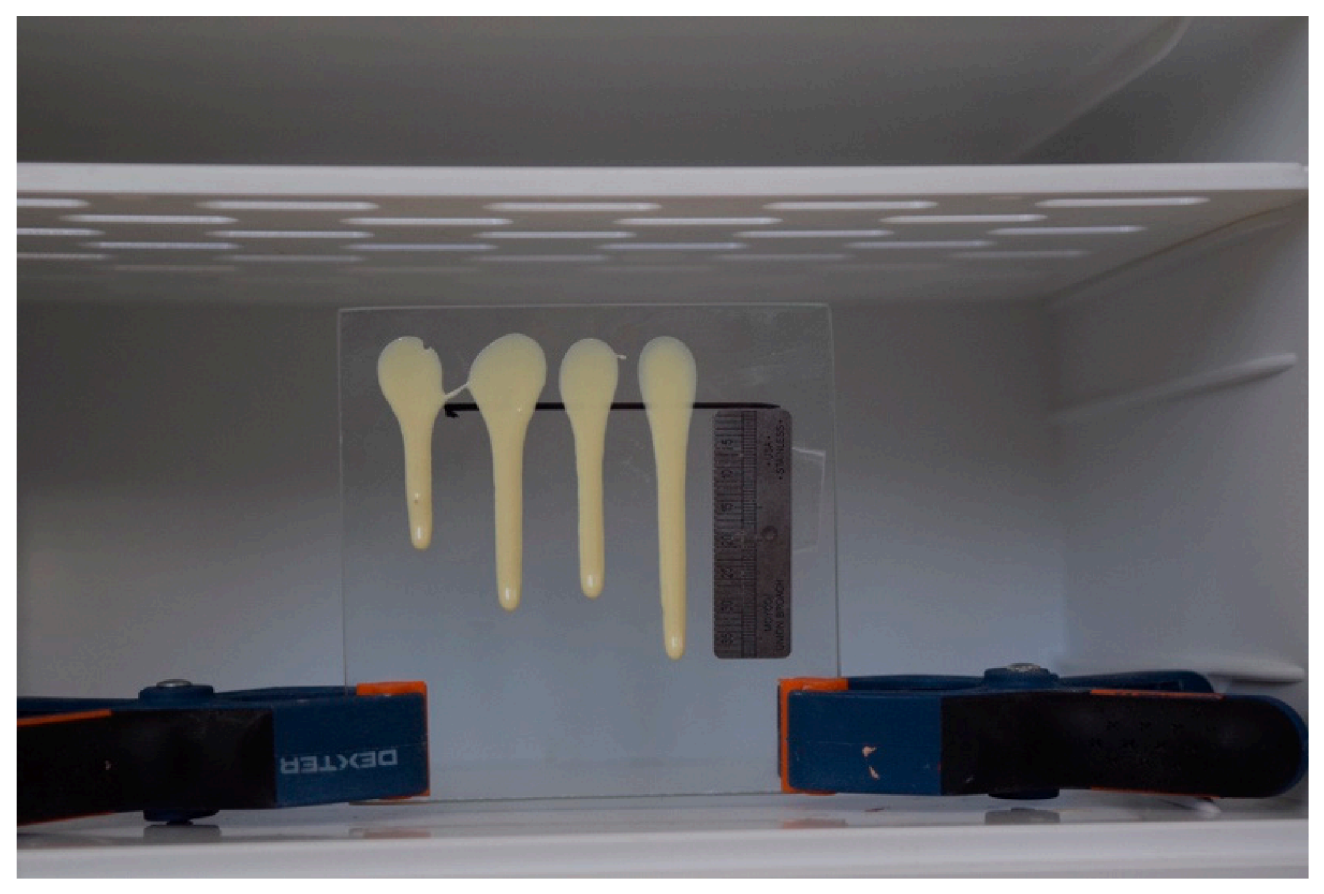
Lateral-flow test setup.

This experiment was conducted at body temperature (37 ± 1 °C). Square glass plates were produced to the required specifications by a glazier: 3 mm thickness, a 4.5 mm square for the base, and a 4.5 mm square for the cover. The thickness of each plate was verified using a micrometre. A volume of 0.1 mL of each material was delivered via micropipette (Eppendorf Reference, adjustable-volume, Eppendorf AG, Hamburg, Germany), at the top of the vertical plate, at the zero point. After 1 min, the distance over which the droplet of each sealer travelled downward was measured (Figure 5). 

## 3. Statistical Analysis

Flow-related measurements were summarized using means and standard deviations, whereas contact angle measurements, which showed appreciable deviation from normality, were summarized using medians and interquartile ranges. Unadjusted global descriptive comparisons were performed using one-way analysis of variance or t-tests for flow-related outcomes and Kruskal–Wallis or Mann–Whitney tests for contact angle summaries, as appropriate. For the two-plate flow experiment, flow expressed as diameter (mm) was analyzed using linear regression. Sealer and temperature (20 °C versus 37 °C) were treated as categorical factors, and the sealer × temperature interaction was included and evaluated using a joint test of the corresponding interaction terms. Lateral-flow and capillary-flow outcomes were analyzed using linear regression with sealer entered as a categorical factor. Additional subgroup analyses were performed for epoxy resin and bioceramic sealers where relevant; in the revised classification, the bioceramic subgroup comprised BC and BC Hi Flow only, whereas MTA Fillapex was treated separately. Contact angle measurements were analyzed using median (quantile) regression. Measurement time (0, 1, 5, and 60 min), measurement side, sealer, substrate (dentin versus gutta-percha), and temperature (20 °C versus 37 °C) were treated as categorical factors. The interaction model evaluated time × substrate, time × sealer, side × sealer, sealer × temperature, and sealer × substrate interactions, with overall interaction effects assessed using joint tests. Because repeated contact angle measurements were obtained from the same experimental units, standard errors and confidence intervals were calculated using the experimental unit ID as the clustering variable; no random effects were fitted. For linear-regression models, assumptions were assessed using residual-versus-fitted plots, normal Q-Q plots, assessment of influential observations, and evaluation of variance heterogeneity. Where heterogeneity of variance was most evident, sensitivity analysis using HC3 heteroskedasticity-robust standard errors did not materially alter the principal conclusions. No formal multiplicity adjustment was applied to secondary model-based contrasts, which were considered exploratory; inference regarding multi-level factors and interactions was based primarily on global/joint tests. Effect estimates are reported with 95% confidence intervals, and detailed results from the principal regression models are provided in Supplementary Tables S1–S13. All tests were two-sided and statistical significance was assessed at the 0.05 level. Analyses were performed using Stata version 14.2 (StataCorp, College Station, TX, USA). *p*-values in the range of 0.05, or less, were considered to be statistically significant [21].

## 4. Results

All results can be downloaded in the supplementary analyses in the link found at the end of the manuscript.

### 4.1. Flow

Through the two-plate-method test, all tested sealers demonstrated greater flow than those suggested both by the ISO Standards (6876/2001, 2nd and 6876/2012 3rd Revision), which call for a minimum of 17 mm, and the 20 mm suggested by the ANSA/ADA Directive [22,23]. The MTA Fillapex sealer had the highest flow, and the GuttaFlow Bioseal flowed the least. The sealers ranked as follow with regard to flow, in ascending order: GuttaFlow Bioseal < BJM < BC Hi Flow < AH Jet < ADSeal < BC < Roth < AH26 < MTA Fillapex (*p* < 0.001) (Table S2). 

GuttaFlow Bioseal sealer yielded the smallest diameter compared to the other sealers. Epoxy-resin-based, bioceramics and MTA Fillapex and ZnO eugenol-based sealers demonstrated larger diameters and thus higher flow. Differences in composition affected the flow of the sealer.

The mean (SD) diameter by sealer and temperature is shown in Table 2. ADSeal, BJM, AH26, and Roth increased their diameter significantly as the temperature increased to body temperature. BC, GuttaFlow Bioseal, and MTA Fillapex were slightly affected by this rise in temperature. 

#### 4.1.1. Flow of Epoxy-Resin-Based Sealers

The ranking of epoxy resin sealers was as follows: BJM < AH JET < ADSeal < AH26 (Table 3, *p* < 0.001).

Among the epoxy resin sealers, ADSeal showed the largest temperature-associated increase in two-plate flow [21.8 mm (95% CI 20.2 to 23.5); *p* < 0.001], whereas AH JET showed the smallest increase [6.6 mm (95% CI 5.0 to 8.2); *p* < 0.001] (Table S5).

#### 4.1.2. Flow of Bioceramics 

The BC Hi Flow presented less flowable compared to BC sealer (*p* < 0.001) (Table 4).

The effect of temperature differed between the two bioceramic sealers (sealer × temperature interaction, *p* = 0.013). For BC, flow increased by 4.1 mm at 37 °C compared with 20 °C (95% CI 2.6 to 5.5; *p* < 0.001), whereas BC Hi Flow showed a smaller, borderline increase of 1.4 mm (95% CI −0.0 to 2.9; *p* = 0.053) (Tables S5 and S13).

### 4.2. Wetting

The median and interquatile range for each sealer on dentin discs and gutta-percha surfaces over 0, 1, 5, and 60 min are shown in Table 5 GuttaFlow Bioseal had the highest contact angle, whereas AH26 had the lowest (*p* < 0.001). 

As for the wetting, the sealers, performed according to the reported medians as follows: AH26 < Roth < ADSeal = BC High Flow < AH JET < MTA Fillapex < BC < BJM < GuttaFlow Bioseal.

#### 4.2.1. Contact Angles for Epoxy-Resin-Based Sealers

Contact angles for epoxy-resin-based sealers are shown in Table 6 (*p* < 0.001).

Epoxy-resin-based sealers were ranked in the ascending order with regard to median angle contact as follows: AH26 < ADSEAL < AH JET < BJM.

#### 4.2.2. Contact Angles for Bioceramic Sealers 

The contact angles for bioceramic-based sealers are shown in Table 7 (*p* < 0.001). By order of increasing median angle, these sealers were ranked as follows: BC Hi Flow < BC.

### 4.3. Contact Angles by Substrate and by Temperature

Contact angles decreased over time. Compared with baseline, the adjusted median differences were −2.2° at 1 min (95% CI −2.7 to −1.8), −4.2° at 5 min (95% CI −4.9 to −3.6), and −6.7° at 60 min (95% CI −7.5 to −5.9; all *p* < 0.001). The effect of substrate varied over time (time × substrate interaction, *p* = 0.003) and by sealer (sealer × substrate interaction, *p* < 0.001), while the effect of temperature varied by sealer (sealer × temperature interaction, *p* < 0.001) (Supplementary Table S5).

### 4.4. Penetration Coefficient

Mean (SD) penetration distances of capillary flow are shown in Table 8 (*p* < 0.001). The highest mean penetration distance was yielded by BJM [8.4 mm (0.8)], whereas GuttaFlow Bioseal presented the lowest [0.4 mm (0.4)]. The sealers were ranked in an increasing order of mean penetration of capillary flow as follows: GuttaFlow Bioseal < MTA Fillapex < AH JET < BC = BC Hi Flow < ADSeal < AH26 = Roth < BJM.

The mean distances (SD), as penetration coefficients, are shown by composition in Table 9 (*p* < 0.001). Zinc oxide eugenol sealer had the highest diameter [7.6 mm (0.7)], whereas GuttaFlow Bioseal had the lowest [0.4 mm (0.4)]. Grouped by composition, the sealers were ranked by mean distance (penetration coefficient) in increasing order as follows: GuttaFlow Bioseal < MTA Fillapex < Bioceramics < epoxy resin < Roth.

#### 4.4.1. Penetration Coefficient of Epoxy-Resin-Based Sealers

The mean distances (SD), as penetration coefficients, of epoxy-resin-based sealers are shown in Table 10 (*p* < 0.001). BJM penetrated the highest distance [8.4 mm (0.8)] and AH JET the lowest [2.9 mm (0.8)]. The epoxy-resin-based sealers were ranked in ascending order of this parameter as follows: AH JET < ADSeal < AH26 < BJM.

#### 4.4.2. Penetration Coefficients of Bioceramic-Based Sealers

BC and BC Hi Flow showed essentially identical mean capillary penetration distances [3.4 (0.5) and 3.4 (0.6) mm, respectively] and did not differ significantly (mean difference 0.0 mm, 95% CI −0.6 to 0.6; *p* = 0.930). (Table 11).

### 4.5. Lateral-Flow/Creeping/Time-Dependent Deformation

Table 12 shows the mean (SD) time-dependent deformation of each sealer. BJM had the highest value [30.9 mm (SD 12.4)] and GuttaFlow Bioseal had the lowest [0.4 mm (SD 0.3)]. In increasing order, the sealers were ranked by lateral flow as follows: GuttaFlow Bioseal < BC < AH JET = MTA Fillapex < BC Hi Flow < ADSeal < Roth < AH26 < BJM.

#### 4.5.1. Time-Dependent Deformation of the Epoxy-Resin-Based Sealers

Table 13 show the mean (SD) time-dependent deformation of the epoxy-resin-based sealers. BJM had the highest mean diameter [30.9 mm (12.4)], whereas AH JET had the lowest [3.3 mm (1.7)]. These sealers were ranked by mean diameter, as a measure of time-dependent deformation, as follows, in increasing order: AH JET < ADSeal < AH26 < BJM.

#### 4.5.2. Time-Dependent Deformation of the Bioceramic Sealers

Table 14 show the mean (SD) time-dependent deformation of bioceramic-based sealers. BC sealer had the lowest value, compared with BC Hi Flow, which had the highest. 

## 5. Discussion

ISO and ADA specifications regarding the flow of endodontic sealers have not required a measurement of viscosity; instead, they use the diameter of a film of sealer between 2 glass plates, which is related to viscosity but easier to measure. Directive ANSI/ADA issued on 2000, requires a minimum diameter of 20 mm, whereas Directive ISO 6876 2nd revision in 2001 and 3rd revision at 2012, requires a 17 mm disc at least [22,23]. Ideal flow requirements that will allow material to flow into all internal spaces have not been established, without extrusion through the apex, producing a seal that is closely adapted to the canal walls, without voids. A study of the rheological properties of endodontic sealers may help identify and achieve the ideal flow pattern. The tendency of a liquid to spread over a solid surface is expressed as the formation of a contact angle [24].

The two-plate method is a simple procedure to perform, yielding flow-related measures, with which variation in the flow of endodontic sealers has been examined [15,25,26,27]. In this study, GuttaFlow Bioseal generated the smallest diameter, likely due to the fast setting of this sealer, whereas MTA Fillapex had the largest, which has also been reported by Sfeir et al. [28], likely due to its high viscosity. Epoxy resin sealers had similar flow behaviours as bioceramic-based sealers. Flow increased with rises in temperature due to the greater viscosity of the sealers, as evidenced with epoxy-resin-based sealers. 

Still, same chemical family, does not mean same behaviour. Farmakis and coworkers in 2012 showed that AH-Plus and TopSeal endodontic sealers, despite having identical formulations (according to the manufacturing company—Densply), presented with different bactericidal properties. Given the fact that they are produced in different countries (AH-Plus in Germany, and TopSeal in Switzerland) and that the raw materials are acquired locally, there might be fine differences in the composition of the raw materials that might significantly affect pharmaceutical and other properties of the final product [29]. This might also be the case in the present study. 

An alternative approach was used in this study to test their rheological behaviour: capillary penetration. This method is performed easily in the laboratory, constituting a better assay for testing sealers than that recommended by the ISO 6876 [22] specification, because the former can demonstrate the likely effect of narrowing root canals or increasing the rate of insertion. It also has the advantage of using a small amount (0.1 mL) of sealer material. Previous studies have shown that flow depends on particle size (molecular dimensions) [16], rate of shear [30], temperature, and rate of setting or setting time [18,31,32].

Furthermore, Lacey et al. demonstrated that rheologically, in a capillary system, flow depends on the internal diameter of the tubes and the rate of insertion and that this effect varies between materials [7].

The measurement of flow of sealers can be complicated by the setting process, wherein the development of chemical crosslinking chains affects the viscosity [33]. This issue was avoided in our study by recording all measurements within the manufacturer’s recommended window. A specific mixing technique, per the manufacturer’s instructions, was followed, and the time from the start of mixing to the end of the test was noted for each material (<3 min).

The rheology of endodontic sealers may be impeded by tapered and curved canals and by the insertion of a gutta-percha point. Flow may be affected by dentine tubules and the presence of a smear layer. However, the results that were acquired from capillary penetration appeared to relate more closely to the clinical situation than those of the two-plate method. Viscosity—and, thus, resistance to flow—is inversely related to eight times the volumetric flow rate (Poisseuille equation).

Lacey et al. have shown that increasing the rate of insertion enhances volumetric flow and thus reduces viscosity for all sealers [7]. Because the internal width of the canals is decreased, volumetric flow lowers, and thus, viscosity rises. Clinicians may have assumed that the flow of sealers will improve by increasing the rate of insertion. However, they must consider not only the sealer that is used but also the width of the root canals. Furthermore, it cannot be assumed that reducing the powder:liquid ratio of a sealer will improve flow, given that this study has shown that flow decreases at a higher rate of insertion [7].

Our choice of method for measuring contact angles is warranted, based on several factors. First, and very importantly, the experiment was performed on human root dentin slices parallel to the root canal—a potentially influential factor that was not taken into account, in any other previous report but simulates clinical conditions. Second, this protocol shows good reliability and comparability with regard to biological variations across patients, with low experimental error [34]. Furthermore, contact angle can vary significantly as a function of the drop size of a liquid, particularly as droplet size increases [35,36]. Notably, our preferred approach allows contact angles to be measured in minute liquid volumes—in this study, 0.1 mL of each sealer—enabling more accurate and reliable contact angle values to be recorded. Finally, the effects of humidity and changes in temperature [37] on the surface tension coefficient values of our sealers were mitigated by the ability of contact angles to be measured under routine laboratory settings.

Our findings thus suggest that the surface free energy of dentin exceeds that of gutta-percha, lending further support to the phenomenon in which, compared with gutta-percha, the forces of the surface energy of dentin surmount those of the surface tension of the sealers in our study. With respect to these results, we conclude that the surface roughness of dentin and the initial incomplete wetting were more influential factors.

All tested sealers exhibited acceptable values with regard to flow, based either on ISO 6786 Standard [22] or the ANSA/ADA Directive [23].recommendations, as mentioned earlier. Flow is an important physical property that indicates the capacity of a sealer to enter and fill spaces that are difficult to access, such as isthmuses, accessory canals, and ramifications [38]. However, a high flow might increase the potential that the material will be extruded toward the periapical region, resulting in direct contact of unset sealer with the biological environment, in turn increasing the potential risk of cytotoxicity and periapical tissue injury [18,39]. In our study, the BC Hi-Flow sealer showed greater flow potential only by lateral-flow/creeping/time-dependent deformation test.

Overall, the contact angle values dentin and gutta-percha after 0, 1, 5, and 60 min were similar for all sealers tested. Critical surface tension is influenced significantly by the underlying routes by which these sealers are synthesized [40]. Yet, despite differences in the synthesis of these sealers, their contact angles approximated each other. Thus, this finding argues for the possibility that the contact angle of a sealer is a function of its synthesis. 

However, the incorporation of silicone into a sealer potentially effects high surface tension forces, impeding the spread of such a compound over a given substrate. In addition to measuring the contact angle of the liquid of a sealer, one study also recorded this value for measured the mixed sealer paste [41]. 

Both measurements were made because the liquid component of a sealer and mixed sealer paste can differ with regard wettability and, in particular, contact angle. We chose specific time points for these measurements in our study (0, 1, and 5 min and 60 min), because they correspond to the start and completion of root canal obturation, respectively. Moreover, because a solid–liquid interaction can yield a range of contact angles—wherein advanced angles can near a maximum and receded angles near a minimum [42,43]—a lone static measurement of contact angle fails to thoroughly describe this interaction [20].

Our dentin substrates were pretreated as optimally as possible to simulate actual clinical conditions. They were prepared out of human root dentin, along the root, a condition that simulates the conditions within the root canal. After being limited to a 5 min sonication to rid the samples of extraorganic compounds (longer times could precipitate a reaction between the water in the sonicator and proteins in the dentin, adversely affecting the outcomes [33]), the specimens were dried at 37 °C. Excessive drying sessions were avoided to prevent dehydration, which can influence the contact angles of sealers [44]. Finally, because certain irrigants, like EDTA, can alter the surface energy and critical surface tension of dentin [41], the surfaces of the dentin discs did not undergo any chemical treatment.

Contact angle, which is measured inside of a liquid droplet where liquid, gas and solid meet, is a significant determinant of a liquid’s wetting behaviour [45] and is influenced by several factors—primarily those of the solid surface, including roughness, contamination, and homogeneity, but also the surface tension of the liquid. Complete wetting, whereby, a liquid spreads entirely over a flat surface, is effected by a contact angle of 0° [45], whereas a contact angle of less than 90° enables partial wetting or hydrophilicity, wherein the liquid spreads out over a surface. In contrast, a liquid is considered non-wetting or hydrophobic if its contact angle on a substrate exceeds 90°, reflected by a liquid beading up into droplets (i.e., the surface repels the liquid). 

These properties are especially important when analyzing the wettability of sealers, which must consider not only the sealers themselves but also the surfaces to which they will be applied—i.e., dentin and gutta-percha. By choosing a sealer with a low contact angle over such substrates, they can overcome surface tension and spread faster throughout these surfaces, especially microcrevices. These properties allow rapid coating and adherence, hopefully resulting in a stronger, more durable bond.

Lateral flow was assessed using vertical glass plates, and penetration co-efficiency was tested with capillary tubes (both at 37°C). Epoxy resin and ZnOE sealers had significantly higher values than the other groups.

Within the epoxy resin sealers, BJM and AH26 showed the highest lateral- and capillary-flow measurements, whereas AH JET and ADSeal generally showed lower values. The magnitude and statistical significance of individual pairwise differences varied between the two assays. These ancillary tests helped us gain a better understanding of the flow behaviour for all sealers, in addition to their wettability and the wetting, spreading, and absorption of liquids.

The physicochemical properties of bioceramic sealers that were observed in this study are consistent with a growing body of literature that has characterized premixed calcium silicate-based formulations. Hamdy et al., in a paper published in 2024, evaluated AH Plus Bioceramic Sealer and Bio-C Sealer against ADSeal resin-based sealer and reported that AH Plus Bioceramic Sealer has the lowest film thickness (20 µm), whereas ADSeal had the highest (80.5 µm) [46]. These findings align with our study’s observation that bioceramic sealers demonstrate comparatively constrained physical behaviour relative to resin-based counterparts, a pattern that extends to their rheological profiles. 

The solubility profile of bioceramic sealers represents a notable limitation that must be contextualized when interpreting their flow behaviour. Quaresma et al. compared AH Plus Bioceramic, Bio-C Sealer, Bio-C Sealer Ion+, and AH Plus across hardening time, dimensional change, solubility, flow, and radiopacity [47]. They reported that AH Plus Bioceramic had the highest solubility among all sealers (12.56%), substantially exceeding the 3% threshold that is stipulated by ANSI/ADA Specification 57, whereas AH Plus was the only sealer to meet this criterion (0.15%). Bio-C Sealer Ion+ and Bio-C Sealer demonstrated the highest flow (59.80 mm and 58.60 mm, respectively); the greatest dimensional shrinkage (−5.19%) was seen with Bio-C Sealer Ion+, whereas AH Plus underwent only marginal expansion (0.03%). These findings contextualize the comparative flow behaviour that we observed in this study: whereas bioceramic sealers, such as EndoSequence BC and BC Hi Flow, demonstrated adequate ISO-compliant two-plate flow, their tendency toward dimensional shrinkage and high solubility after setting, may compromise the long-term hermetic seal, particularly in cases in which extensive calcium ion release leads to volumetric loss within the set material. In 2024, Raman and Camilleri similarly reported that all three hydraulic cement-based sealers that they evaluated (BC Universal sealer, Totalfill BC sealer, and AH Plus Bioceramic) exceeded the ISO 6876:2012 solubility limit of 3%, with AH Plus Bioceramic disintegrating entirely after 7-day immersion in Hank’s balanced salt solution—a worrying finding with direct implications for the longevity of the root canal seal in a physiological environment [48], which is consistent with a systematic review by Silva et al. (2021), who reported concerning results regarding solubility for calcium silicate-based sealers in clinical use [49].

The characterization that was undertaken by Raman and Camilleri [48] provides additional microstructural context for the physical properties that we observed in our study. Using scanning electron microscopy and X-ray diffraction, they showed that AH Plus Bioceramic sealer is characterized by the finest particle size and highest zirconium dioxide content (50–75%) among the hydraulic sealers that were tested, which accounts for its notably thin film thickness (13.3 µm). Totalfill BC sealer, the composition of which more closely resembles that of EndoSequence BC (the same formulation under a different brand name in some markets), had a higher degree of cement hydration and a more moderate film thickness (46.7 µm), consistent with the flow and wettability profile of EndoSequence BC in the present study. The divergent calcium ion release profiles that were reported by Raman and Camilleri further highlight the compositional heterogeneity within the “bioceramic” category that is likely to underlie the rheological differences that were observed here [47,49]. Collectively, these characterization data underscore that generalization of the behaviour of bioceramic sealers as a class is inappropriate without accounting for specific formulation variables.

Considering the limitations of this in vitro study—i.e., no load was applied to any of the test materials—we also found that the wetting behaviour of sealers (according to reported differences and median-regression results), to dentin and gutta-percha do not behave similarly. We acknowledge that behaviour of the sealers in this study could shift during obturation of a root canal system if lateral or vertical compaction pressure is exerted. GuttaFlow Bioseal must be compacted vertically as much as possible so that it achieves sufficient flow within the root canal system, particularly in its crevices. Yet, unlike other sealers, other than pressure during vertical compaction at the conclusion of the procedure, no lateral compaction pressure is administered when GuttaFlow Bioseal is used for obturation.

The properties that were examined in this study have an influential but not completely decisive effect on the clinical behaviours of sealers. To this end, future studies should examine other physical characteristics of a material, such as its viscosity, film thickness, solubility, and biocompatibility, to generate more robust and definitive conclusions. Furthermore, one should keep in mind that in vitro studies cannot be implemented directly for clinical applications, despite their usefulness. It should also be noted that few papers have been published on the rheological properties of endodontic sealers since early 2020 to late 2025 [4,47,48,49,50], thus preventing a more robust and fruitful discussion of this topic.

Finally, the findings of this study suggest that the ANSA/DIN/ISO directives for the testing of biomaterials should be modified to advise that future in vitro research be performed at body temperature, given that this parameter significantly affects the results.

## 6. Conclusions

Within the limitations of this study, the rheological properties of endodontic sealers depend on the nature of the substrate, the composition of the sealers, and substrate temperature. More specifically, higher temperature resulted in increased flow for both epoxy-resin-based and ZnO eugenol-based sealers, whereas Bioceramics, MTA Fillapex, and GuttaFlow Bioseal did not follow this trend. At 20 °C, gutta-percha substrate resulted in lower contact angles than dentin, but at body temperature, the differences in contact angle lessened between substrates. Body temperature significantly influenced the outcome.

## Figures and Tables

**Table 1:** The sealers used along with useful information.

Chemical Base	Sealer	Manufacturer	Form Type
Zinc oxide eugenol	Roth 801	Roth International, Chicago, IL, USA	Powder/liquid
Epoxy resin	AH26 silver-free	Dentsply De Trey GmbH, Konstanz, Germany	Powder/liquid
Epoxy resin	AH JET	Dentsply De Trey GmbH, Konstanz, Germany	Dual syringe automix
Epoxy resin	BJM Root Canal Sealer	BJM Laboratories, Or-Yehuda, Israel	Dual syringe automix
Epoxy resin	ADSeal	Meta Biomed Co., Cheongju, Korea	Paste/paste dual syringe
Salicylate cement	MTA Fillapex	Angelus Ind. Prod. Odontológicos S/A, Londrina, PR, Brazil	Dual syringe automix
Calcium silicate cement	EndoSequence BC	Brasseler, Savannah, GA, USA	Single-syringe delivery system
Calcium silicate cement	EndoSequence BC Hi Flow	Brasseler, Savannah, GA, USA	Single-syringe delivery system
Silicone and calcium silicate cement	GuttaFlow Bioseal	Coltene/Whaledent, Altstätten, Switzerland	Dual syringe automix

**Table 2:** Diameter by sealer and temperature.

Sealer/Temperature	N	Diameter (mm)
		Mean (SD)
*MTA Fillapex*—20 °C	6	61.3 (1.9)
*MTA Fillapex*—37 °C	6	67.2 (1.7)
*GuttaFlow Bioseal*—20 °C	6	29.0 (0.6)
*GuttaFlow Bioseal*—37 °C	6	30.7 (1.2)
*BJM*—20 °C	6	29.5 (1.0)
*BJM*—37 °C	6	46.8 (1.9)
*BC*—20 °C	6	46.2 (0.8)
*BC*—37 °C	6	50.3 (1.2)
*BC Hi Flow*—20 °C	6	39.8 (1.1)
*BC Hi Flow*—37 °C	6	41.2 (1.5)
*ADSeal*—20 °C	6	30.7 (2.2)
*ADSeal*—37 °C	6	52.5 (1.0)
*AH26*—20 °C	6	47.7 (0.8)
*AH26*—37 °C	6	65.5 (1.0)
*Roth*—20 °C	6	44.2 (1.9)
*Roth*—37 °C	6	62.0 (3.2)
***Overall***	96	46.5 (13.2)

**Table 3:** Diameter by epoxy sealer, *p* < 0.001.

Sealer	N	Diameter (mm)
		Mean (SD)
*AH26*	12	56.7 (9.4)
*AH JET*	12	41.2 (3.6)
*BJM*	12	38.2 (9.1)
*ADSeal*	12	41.6 (11.5)
***Overall***	48	44.4 (11.3)

**Table 4:** Diameter by bioceramic sealer, *p* < 0.001.

Sealer	N	Diameter (mm)
		Mean (SD)
*BC*	12	48.3 (2.3)
*BC Hi Flow*	12	40.5 (1.5)
***Total***	24	44.4 (4.4)

**Table 5:** Contact angle by composition of sealer, *p* < 0.001.

Sealers (Grouped)	N	Angle
		Median (IQR)
*Epoxy resin*	2184	64.1 (55.7, 70.8)
*Bioceramics*	944	71.2 (63.4, 78.7)
*GuttaFlow Bioseal*	640	78.8 (74.5, 81.9)
*Roth*	640	62.3 (54.2, 67.8)
*MTA Fillapex*	640	72.1 (64.5, 79.3)
***Overall***	5048	67.3 (60.1, 76.8)

**Table 6:** Contact angles by epoxy-resin-based sealer, *p* < 0.001.

Sealer	N	Angle
		Median (IQR)
*AH26*	640	54.0 (45.7, 61.9)
*AH JET*	264	65.0 (60.2, 68.7)
*BJM*	640	76.2 (69.1, 81.3)
*ADSeal*	640	63.0 (57.1, 66.5)
***Overall***	2184	64.1 (55.7, 70.8)

**Table 7:** Contact angles by bioceramic sealer, *p* < 0.001.

Sealer	N	Angle
		Median (IQR)
*BC*	640	75.5 (68.5, 80.7)
*BC Hi Flow*	304	63.0 (55.2, 67.7)
***Total***	944	71.2 (63.4, 78.7)

**Table 8:** Penetration coefficient by sealer, *p* < 0.001.

Sealer	N	Diameter (mm)
		Mean (SD)
*AH26*	8	7.6 (0.5)
*AH JET*	8	2.9 (0.8)
*BJM*	8	8.4 (0.8)
*ADSeal*	8	3.7 (0.5)
*MTA Fillapex*	8	2.4 (0.6)
*BC*	8	3.4 (0.5)
*BC Hi Flow*	8	3.4 (0.6)
*GuttaFlow Bioseal*	8	0.4 (0.4)
*Roth*	8	7.6 (0.7)
***Overall***	72	4.4 (2.7)

**Table 9:** Distance penetration coefficient by composition of sealer, *p* < 0.001.

Sealers (Grouped)	N	Penetration (mm)
		Mean (SD)
*Epoxy resin*	32	5.6 (2.5)
*Bioceramic*	16	3.4 (0.5)
*MTA Fillapex*	8	2.4 (0.6)
*GuttaFlow Bioseal*	8	0.4 (0.4)
*Roth*	8	7.6 (0.7)
***Overall***	72	4.4 (2.7)

**Table 10:** Distance penetration coefficient by sealer, *p* < 0.001.

Sealer	N	Distance (mm)
		Mean (SD)
*AH26*	8	7.6 (0.5)
*AH JET*	8	2.9 (0.8)
*BJM*	8	8.4 (0.8)
*ADSeal*	8	3.7 (0.5)
***Overall***	32	5.6 (2.5)

**Table 11:** Distance penetration coefficient by bioceramic-based sealer, *p* = 0.930.

r	N	Diameter (mm)
Sealer		Mean (SD)
*BC*	8	3.4 (0.5)
*BC Hi Flow*	8	3.4 (0.6)
***Overall***	16	3.4 (0.5)

**Table 12:** Distance lateral-flow/creeping/time-dependent deformation by sealer, *p* < 0.001.

Sealer	N	Distance (mm)
		Mean (SD)
*AH26*	8	26.2 (11.9)
*AH JET*	8	3.3 (1.7)
*BJM*	8	30.9 (12.4)
*ADSeal*	8	16.7 (5.8)
*MTA FILLAPEX*	8	3.3 (0.7)
*BC*	8	1.6 (1.9)
*BC Hi Flow*	8	6.8 (1.3)
*GuttaFlow Bioseal*	8	0.4 (0.3)
*Roth*	8	19.9 (4.5)
***Overall***	72	12.1 (12.5)

**Table 13:** Lateral-flow/creeping/time-dependent deformation of epoxy-resin-based sealers, *p* < 0.001.

Sealer	N	Distance (mm)
		Mean (SD)
*AH26*	8	26.2 (11.9)
*AH JET*	8	3.3 (1.7)
*BJM*	8	30.9 (12.4)
*ADSeal*	8	16.7 (5.8)
***Overall***	32	19.3 (13.8)

**Table 14:** Distance lateral-flow/creeping/time-dependent deformation by bioceramic-based sealer. *p* < 0.001.

Sealer	N	Distance (mm)
		Mean (SD)
*BC*	8	1.6 (1.9)
*BC Hi Flow*	8	6.8 (1.3)
***Overall***	16	4.2 (3.1)

## Data Availability

The original contributions presented in this study are included in the article/Supplementary Material. Further inquiries can be directed to the corresponding authors.

## Supplementary Materials

The following supporting information can be downloaded at: https://www.mdpi.com/article/10.3390/jfb17090448/s1, Table S1: Overall linear regression analysis of two-plate flow (diameter, mm) according to sealer and temperature; Table S2: Linear regression analysis of two-plate flow among epoxy-resin sealers; Table S3: Revised linear regression analysis of two-plate flow among bioceramic sealers (BC and BC High Flow only); Table S4: Overall median regression analysis of contact angle without interaction terms; Table S5: Overall median regression analysis of contact angle including two-way interaction terms; Table S6: Revised median regression analysis of contact angle among bioceramic sealers without interaction terms; Table S7: Revised median regression analysis of contact angle among bioceramic sealers including two-way interaction terms; Table S8: Overall linear regression analysis of lateral flow/creeping/time-dependent deformation according to sealer; Table S9: Linear regression analysis of lateral flow among epoxy-resin sealers; Table S10: Revised linear regression analysis of lateral flow among bioceramic sealers; Table S11: Overall linear regression analysis of capillary flow/penetration distance according to sealer; Table S12: Linear regression analysis of capillary flow among epoxy-resin sealers; Table S13: Revised linear regression analysis of capillary flow among bioceramic sealers.
