# Supplementary material for: Rheological Properties of Nine Commercially Available Endodontic Sealers by Substrate and Temperature: A Comparative In Vitro Study
Source: J Funct Biomater. 2026 Sep 5;17(9):448. doi: 10.3390/jfb17090448 (PMC13608539; doi:10.3390/jfb17090448)
Supplement: Supplementary file 1 [file jfb-17-00448-s001.zip › jfb-4493358-supplementary.pdf]

Supplementary Statistical Results

The tables below provide regression coefficients, 95% confidence intervals (CIs), and nominal (unadjusted) p-values from the principal statistical models and key subgroup analyses. Coefficients from linear regression represent mean differences, whereas coefficients from median regression represent differences in the conditional median. Reference categories are identified in the tables. In accordance with the revised material classification, the bioceramic subgroup includes BC and BC High Flow only; MTA Fillapex is treated separately.

Table S1. Overall linear regression analysis of two-plate flow (diameter, mm) according to sealer and temperature.

Factor / term	Coefficient	95% CI	p-value
**Sealer**			
AH26 (reference)	0		
AH JET	-9.8	(-11.6, -8.0)	<0.001
BJM	-18.2	(-20.0, -16.4)	<0.001
ADSeal	-17.1	(-18.9, -15.3)	<0.001
MTA Fillapex	13.6	(11.8, 15.4)	<0.001
BC	-1.5	(-3.3, 0.3)	0.099
BC High Flow	-7.9	(-9.7, -6.1)	<0.001
GuttaFlow Bioseal	-18.8	(-20.6, -17.0)	<0.001
Roth	-3.6	(-5.4, -1.8)	<0.001
**Temperature**			
20°C (reference)	0		
37°C	17.8	(16.0, 19.6)	<0.001
**Sealer × temperature interaction**			
AH JET × 37°C	-11.3	(-13.8, -8.7)	<0.001
BJM × 37°C	-0.6	(-3.1, 1.9)	0.647
ADSeal × 37°C	4.0	(1.5, 6.5)	0.002
MTA Fillapex × 37°C	-12.0	(-14.5, -9.5)	<0.001
BC × 37°C	-13.8	(-16.3, -11.2)	<0.001
BC High Flow × 37°C	-16.4	(-18.9, -13.9)	<0.001
GuttaFlow Bioseal × 37°C	-16.1	(-18.6, -13.6)	<0.001
Roth × 37°C	-0.0	(-2.5, 2.5)	1.000
Constant	47.7	(46.5, 49.0)	<0.001

Reference combination: AH26 at 20°C. Overall sealer × temperature interaction: p<0.001. For the revised BC/BC High Flow subgroup-specific inference used in the manuscript, see Table S3.

Table S2. Linear regression analysis of two-plate flow among epoxy-resin sealers.

Factor / term	Coefficient	95% CI	p-value
**Sealer**			
AH26 (reference)	0		
AH JET	-9.8	(-11.5, -8.2)	<0.001
BJM	-18.2	(-19.8, -16.5)	<0.001
ADSeal	-17.1	(-18.7, -15.5)	<0.001
**Temperature**			
20°C (reference)	0		
37°C	17.8	(16.2, 19.5)	<0.001
**Sealer × temperature interaction**			
AH JET × 37°C	-11.3	(-13.5, -9.0)	<0.001
BJM × 37°C	-0.6	(-2.9, 1.7)	0.610
ADSeal × 37°C	4.0	(1.7, 6.3)	0.001

Factor / term	Coefficient	95% CI	p-value
Constant	47.8	(46.6, 48.9)	<0.001

Reference combination: AH26 at 20°C. Overall sealer × temperature interaction: p<0.001.

Model-derived temperature effects within epoxy-resin sealers:

Sealer	Estimated difference (37°C - 20°C), mm	95% CI	p-value
AH26	17.8	(16.2, 19.5)	<0.001
AH JET	6.6	(5.0, 8.2)	<0.001
BJM	17.3	(15.6, 18.9)	<0.001
ADSeal	21.8	(20.2, 23.5)	<0.001

Table S3. Revised linear regression analysis of two-plate flow among bioceramic sealers (BC and BC High Flow only).

Factor / term	Coefficient	95% CI	p-value
**Sealer**			
BC (reference)	0		
BC High Flow	-6.4	(-7.9, -5.0)	<0.001
**Temperature**			
20°C (reference)	0		
37°C	4.1	(2.6, 5.5)	<0.001
**Sealer × temperature interaction**			
BC High Flow × 37°C	-2.7	(-4.7, -0.6)	0.013
Constant	46.2	(45.2, 47.3)	<0.001

Reference combination: BC at 20°C. Overall sealer × temperature interaction: p=0.013. This revised subgroup model is the source for the bioceramic-specific statements in the manuscript.

Model-derived temperature effects within the revised bioceramic subgroup:

Sealer	Estimated difference (37°C - 20°C), mm	95% CI	p-value
BC	4.1	(2.6, 5.5)	<0.001
BC High Flow	1.4	(-0.0, 2.9)	0.053

The subgroup-specific standard errors and p-values may differ slightly from contrasts derived from the overall nine-sealer model because residual variance is estimated within the subgroup model.

Table S4. Overall median regression analysis of contact angle without interaction terms.

Factor / term	Coefficient	95% CI	p-value
**Time**			
0 min (reference)	0		
1 min	-2.2	(-2.7, -1.8)	<0.001
5 min	-4.2	(-4.9, -3.6)	<0.001
60 min	-6.7	(-7.5, -5.9)	<0.001
**Side**			
Left (reference)	0		
Right	-0.9	(-1.6, -0.1)	0.029
**Sealer**			
AH26 (reference)	0		
AH JET	12.3	(9.4, 15.3)	<0.001
BJM	22.3	(19.6, 25.1)	<0.001
ADSeal	8.9	(6.0, 11.7)	<0.001
MTA Fillapex	19.5	(16.8, 22.3)	<0.001
BC	22.5	(19.9, 25.1)	<0.001
BC High Flow	9.8	(6.7, 12.9)	<0.001
GuttaFlow Bioseal	24.8	(22.3, 27.3)	<0.001
Roth	9.0	(6.0, 12.0)	<0.001
**Substrate**			
Dentin (reference)	0		
Gutta-percha	4.5	(3.3, 5.6)	<0.001
**Temperature**			

Factor / term	Coefficient	95% CI	p-value
20°C (reference)	0		
37°C	-1.3	(-2.4, -0.1)	0.027
Constant	55.4	(52.9, 57.8)	<0.001

Cluster-adjusted inference was used at the experimental-unit ID level.

Table S5. Overall median regression analysis of contact angle including two-way interaction terms.

Factor / term	Coefficient	95% CI	p-value
**Time**			
0 min (reference)	0		
1 min	-4.4	(-6.6, -2.3)	<0.001
5 min	-7.7	(-10.6, -4.8)	<0.001
60 min	-11.3	(-14.2, -8.4)	<0.001
**Substrate**			
Dentin (reference)	0		
Gutta-percha	7.5	(2.6, 12.4)	0.003
**Time × substrate interaction**			
1 min × Gutta-percha	0.4	(-0.5, 1.3)	0.359
5 min × Gutta-percha	1.5	(0.3, 2.8)	0.019
60 min × Gutta-percha	2.7	(1.2, 4.1)	<0.001
**Sealer**			
AH26 (reference)	0		
AH JET	18.1	(11.0, 25.2)	<0.001
BJM	17.2	(9.8, 24.5)	<0.001
ADSeal	10.8	(4.2, 17.4)	0.001
MTA Fillapex	23.6	(16.4, 30.7)	<0.001
BC	21.7	(15.1, 28.2)	<0.001
BC High Flow	14.9	(8.4, 21.3)	<0.001
GuttaFlow Bioseal	24.7	(18.4, 31.1)	<0.001
Roth	11.9	(5.1, 18.7)	0.001
**Time × sealer interaction**			
1 min × AH JET	0.6	(-2.2, 3.5)	0.657
1 min × BJM	2.5	(0.2, 4.9)	0.031
1 min × ADSeal	2.6	(0.3, 4.9)	0.028
1 min × MTA Fillapex	2.1	(-0.1, 4.4)	0.066
1 min × BC	2.7	(0.6, 4.8)	0.013
1 min × BC High Flow	0.8	(-2.3, 4.0)	0.597
1 min × GuttaFlow Bioseal	2.9	(0.8, 5.1)	0.007
1 min × Roth	1.3	(-1.0, 3.7)	0.259
5 min × AH JET	0.6	(-4.6, 5.7)	0.826
5 min × BJM	3.2	(0.1, 6.2)	0.040
5 min × ADSeal	2.7	(-0.6, 5.9)	0.105
5 min × MTA Fillapex	3.3	(-0.0, 6.6)	0.051
5 min × BC	4.6	(1.9, 7.3)	0.001
5 min × BC High Flow	0.8	(-2.3, 4.0)	0.597
5 min × GuttaFlow Bioseal	4.5	(1.7, 7.3)	0.002
5 min × Roth	1.4	(-2.2, 5.0)	0.437
60 min × AH JET	-1.5	(-7.2, 4.2)	0.613
60 min × BJM	4.1	(0.9, 7.3)	0.013
60 min × ADSeal	3.2	(-0.0, 6.4)	0.052
60 min × MTA Fillapex	2.9	(-0.2, 6.1)	0.063
60 min × BC	5.4	(2.6, 8.3)	<0.001
60 min × BC High Flow	-1.1	(-4.2, 2.0)	0.492
60 min × GuttaFlow Bioseal	5.8	(2.9, 8.7)	<0.001
60 min × Roth	2.3	(-1.7, 6.3)	0.258
**Side**			
Left (reference)	0		
Right	-1.4	(-3.6, 0.7)	0.178

Factor / term	Coefficient	95% CI	p-value
**Side × sealer interaction**			
Right × AH JET	0.2	(-4.0, 4.4)	0.935
Right × BJM	3.6	(-0.2, 7.4)	0.065
Right × ADSeal	2.3	(-0.5, 5.1)	0.110
Right × MTA Fillapex	-1.4	(-4.6, 1.9)	0.415
Right × BC	-2.9	(-6.5, 0.7)	0.111
Right × BC High Flow	-0.3	(-3.0, 2.5)	0.860
Right × GuttaFlow Bioseal	0.5	(-2.3, 3.3)	0.738
Right × Roth	0.3	(-2.6, 3.2)	0.840
**Temperature**			
20°C (reference)	0		
37°C	-0.2	(-4.2, 3.9)	0.943
**Sealer × temperature interaction**			
AH JET × 37°C	-6.3	(-13.7, 1.0)	0.088
BJM × 37°C	1.5	(-3.5, 6.4)	0.565
ADSeal × 37°C	1.4	(-3.6, 6.3)	0.593
MTA Fillapex × 37°C	-5.0	(-11.0, 0.9)	0.099
BC × 37°C	1.7	(-3.2, 6.6)	0.489
BC High Flow × 37°C	-6.0	(-11.3, -0.8)	0.025
GuttaFlow Bioseal × 37°C	1.5	(-3.2, 6.1)	0.539
Roth × 37°C	-4.3	(-9.6, 1.0)	0.113
**Sealer × substrate interaction**			
AH JET × Gutta-percha	-4.8	(-13.4, 3.8)	0.276
BJM × Gutta-percha	1.0	(-4.7, 6.7)	0.737
ADSeal × Gutta-percha	-9.5	(-15.2, -3.9)	0.001
MTA Fillapex × Gutta-percha	-4.4	(-11.0, 2.2)	0.192
BC × Gutta-percha	-3.3	(-8.8, 2.2)	0.238
BC High Flow × Gutta-percha	-3.8	(-9.9, 2.3)	0.225
GuttaFlow Bioseal × Gutta-percha	-6.2	(-11.5, -0.9)	0.022
Roth × Gutta-percha	-3.2	(-9.2, 2.8)	0.299
Constant	54.8	(48.8, 60.9)	<0.001

Cluster-adjusted inference was used at the experimental-unit ID level. Omnibus interaction tests: time × substrate p=0.003; side × sealer p=0.050; sealer × temperature p<0.001; time × sealer p<0.001; sealer × substrate p<0.001.

Table S6. Revised median regression analysis of contact angle among bioceramic sealers without interaction terms.

Factor / term	Coefficient	95% CI	p-value
**Time**			
0 min (reference)	0		
1 min	-2.1	(-2.9, -1.3)	<0.001
5 min	-3.4	(-4.5, -2.3)	<0.001
60 min	-6.6	(-8.1, -5.1)	<0.001
**Side**			
Left (reference)	0		
Right	-2.7	(-5.0, -0.4)	0.023
**Sealer**			
BC (reference)	0		
BC High Flow	-12.5	(-15.0, -10.0)	<0.001
**Substrate**			
Dentin (reference)	0		
Gutta-percha	4.6	(2.1, 7.1)	<0.001
**Temperature**			
20°C (reference)	0		
37°C	-1.7	(-4.2, 0.8)	0.184
Constant	78.4	(75.5, 81.3)	<0.001

Bioceramic subgroup: BC and BC High Flow only. Cluster-adjusted inference was used at the experimental-unit ID level.

Table S7. Revised median regression analysis of contact angle among bioceramic sealers including two-way interaction terms.

Factor / term	Coefficient	95% CI	p-value
**Time**			
0 min (reference)	0		
1 min	-1.6	(-2.5, -0.7)	0.001
5 min	-2.9	(-4.0, -1.8)	<0.001
60 min	-5.2	(-6.5, -3.9)	<0.001
**Substrate**			
Dentin (reference)	0		
Gutta-percha	4.3	(1.5, 7.1)	0.002
**Time × substrate interaction**			
1 min × Gutta-percha	0.4	(-0.8, 1.6)	0.525
5 min × Gutta-percha	0.6	(-0.9, 2.1)	0.433
60 min × Gutta-percha	2.0	(0.4, 3.6)	0.013
**Sealer**			
BC (reference)	0		
BC High Flow	-7.0	(-10.7, -3.3)	<0.001
**Time × sealer interaction**			
1 min × BC High Flow	-1.7	(-3.2, -0.2)	0.026
5 min × BC High Flow	-3.5	(-5.4, -1.6)	<0.001
60 min × BC High Flow	-6.8	(-8.7, -4.9)	<0.001
**Side**			
Left (reference)	0		
Right	-4.4	(-7.2, -1.6)	0.002
**Side × sealer interaction**			
Right × BC High Flow	2.9	(-0.5, 6.3)	0.094
**Temperature**			
20°C (reference)	0		
37°C	1.5	(-1.2, 4.2)	0.284
**Sealer × temperature interaction**			
BC High Flow × 37°C	-8.2	(-12.5, -3.9)	<0.001
**Sealer × substrate interaction**			
BC High Flow × Gutta-percha	-0.3	(-4.9, 4.3)	0.898
Constant	76.4	(73.8, 79.0)	<0.001

Bioceramic subgroup: BC and BC High Flow only. Cluster-adjusted inference was used at the experimental-unit ID level. Omnibus interaction tests: time × substrate p=0.100; side × sealer p=0.094; sealer × temperature p<0.001; time × sealer p<0.001; sealer × substrate p=0.898.

Table S8. Overall linear regression analysis of lateral flow/creeping/time-dependent deformation according to sealer.

Factor / term	Coefficient	95% CI	p-value
**Sealer**			
AH26 (reference)	0		
AH JET	-22.9	(-29.2, -16.6)	<0.001
BJM	4.7	(-1.6, 11.0)	0.142
ADSeal	-9.5	(-15.8, -3.2)	0.004
MTA Fillapex	-22.9	(-29.2, -16.6)	<0.001
BC	-24.6	(-30.9, -18.3)	<0.001
BC High Flow	-19.4	(-25.7, -13.1)	<0.001
GuttaFlow Bioseal	-25.8	(-32.1, -19.5)	<0.001
Roth	-6.3	(-12.6, -0.0)	0.050
Constant	26.2	(21.7, 30.6)	<0.001

AH26 is the reference category. In the HC3 sensitivity analysis, the overall sealer effect remained highly significant (F[8,63]=59.50, p<0.001).

Table S9. Linear regression analysis of lateral flow among epoxy-resin sealers.

Factor / term	Coefficient	95% CI	p-value
**Sealer**			
AH26 (reference)	0		
AH JET	-22.9	(-32.2, -13.6)	<0.001
BJM	4.7	(-4.6, 14.0)	0.312

Factor / term	Coefficient	95% CI	p-value
ADSeal	-9.5	(-18.8, -0.2)	0.046
Constant	26.2	(19.6, 32.8)	<0.001

AH26 is the reference category.

Table S10. Revised linear regression analysis of lateral flow among bioceramic sealers.

Factor / term	Coefficient	95% CI	p-value
**Sealer**			
BC (reference)	0		
BC High Flow	5.1	(3.4, 6.9)	<0.001
Constant	1.6	(0.4, 2.9)	0.014

Bioceramic subgroup: BC and BC High Flow only. BC is the reference category.

Table S11. Overall linear regression analysis of capillary flow/penetration distance according to sealer.

Factor / term	Coefficient	95% CI	p-value
**Sealer**			
AH26 (reference)	0		
AH JET	-4.7	(-5.3, -4.1)	<0.001
BJM	0.7	(0.1, 1.3)	0.019
ADSeal	-4.0	(-4.6, -3.4)	<0.001
MTA Fillapex	-5.2	(-5.8, -4.6)	<0.001
BC	-4.2	(-4.8, -3.6)	<0.001
BC High Flow	-4.2	(-4.8, -3.6)	<0.001
GuttaFlow Bioseal	-7.2	(-7.8, -6.6)	<0.001
Roth	0.0	(-0.6, 0.6)	1.000
Constant	7.6	(7.2, 8.1)	<0.001

AH26 is the reference category.

Table S12. Linear regression analysis of capillary flow among epoxy-resin sealers.

Factor / term	Coefficient	95% CI	p-value
**Sealer**			
AH26 (reference)	0		
AH JET	-4.7	(-5.4, -4.0)	<0.001
BJM	0.7	(0.0, 1.4)	0.038
ADSeal	-4.0	(-4.7, -3.3)	<0.001
Constant	7.6	(7.1, 8.1)	<0.001

AH26 is the reference category.

Table S13. Revised linear regression analysis of capillary flow among bioceramic sealers.

Factor / term	Coefficient	95% CI	p-value
**Sealer**			
BC (reference)	0		
BC High Flow	0.0	(-0.6, 0.6)	0.930
Constant	3.4	(3.0, 3.8)	<0.001

Bioceramic subgroup: BC and BC High Flow only. BC is the reference category. The BC High Flow versus BC difference is 0.0 mm (95% CI -0.6 to 0.6; p=0.930).
